# Molecular Phylogeny and Morphology of *Amphisphaeria* (= *Lepteutypa*) (Amphisphaeriaceae)

**DOI:** 10.3390/jof6030174

**Published:** 2020-09-17

**Authors:** Milan C. Samarakoon, Sajeewa S. N. Maharachchikumbura, Jian-Kui (Jack) Liu, Kevin D. Hyde, Itthayakorn Promputtha, Marc Stadler

**Affiliations:** 1 Department of Biology, Faculty of Science, Chiang Mai University, Chiang Mai 50200, Thailand; samarakoon_m@cmu.ac.th; 2 Research Center in Bioresources for Agriculture, Industry and Medicine, Chiang Mai University, Chiang Mai 50200, Thailand; 3 Center of Excellence in Fungal Research, Mae Fah Luang University, Chiang Rai 57100, Thailand; coe-fungal@mfu.ac.th; 4 School of Life Science and Technology, University of Electronic Science and Technology of China, Chengdu 611731, China; sajeewa83@yahoo.com (S.S.N.M.); liujiankui@uestc.edu.cn (J.-K.L.); 5 Graduate School, Chiang Mai University, Chiang Mai 50200, Thailand; 6 Innovative Institute of Plant Health, Zhongkai University of Agriculture and Engineering, Haizhu District, Guangzhou 510225, China; 7 Department of Microbial Drugs, Helmholtz-Zentrum für Infektionsforschung GmbH, Inhoffenstrasse 7, 38124 Braunschweig, Germany

**Keywords:** 3 new taxa, 6 new combinations, asexual morph, Sporocadaceae, taxonomy

## Abstract

Amphisphaeriaceous taxa (fungi) are saprobes on decaying wood in terrestrial, mangrove, and freshwater habitats. The generic boundaries of the family have traditionally been based on morphology, and the delimitation of genera has always been challenging. *Amphisphaeria* species have clypeate ascomata and 1-septate ascospores and a coelomycetous asexual morph. *Lepteutypa* is different from *Amphisphaeria* in having eutypoid stromata and more than 1-septate ascospores. These main characters have been used for segregation of *Lepteutypa* from *Amphisphaeria* for a long time. However, the above characters are overlapping among *Amphisphaeria* and *Lepteutypa* species. Therefore, here we synonymized *Lepteutypa* under *Amphisphaeria* based on holomorphic morphology and multigene phylogeny. Further, our cluster analysis reveals the relationship between seven morphological traits among *Amphisphaeria*/*Lepteutypa* species and suggests those morphologies are not specific to either genus. Three new species (i.e., *Amphisphaeria camelliae*, *A. curvaticonidia,* and *A. micheliae*) are introduced based on morphology and LSU-ITS-RPB2-TUB2 phylogenies. Furthermore, the monotypic genus *Trochilispora*, which had been accepted in Amphisphaeriaceae, is revisited and synonymized under *Hymenopleella* and placed in Sporocadaceae.

## 1. Introduction

*Amphisphaeria*, the type genus of Amphisphaeriaceae, was introduced by Cesati and De Notaris [1]. *Amphisphaeria* has immersed, clypeate, globose, periphysate ostiolate ascomata; visible as raised, blackened, circular dots on the host surface; and several peridial layers with inner hyaline and outer brown cells; filamentous, septate, flexuous paraphyses; 8-spored, cylindrical asci with J+ or J−, discoid, tubular or wedge-shaped apical ring; and 1-septate, ellipsoidal, brown ascospores [1,2]. The coelomycetous asexual morph has solitary or aggregated, globose, dark brown conidiomata with a thick-walled peridium, septate, branched, hyaline conidiophores, elongated, conical, thin-walled, septate, hyaline, annellidic conidiogenous cells and hyaline, elongate-fusiform, 1-celled, smooth-walled conidia [3]. Wang et al. [2] revised *Amphisphaeria* based on herbarium specimens and accepted 12 species. Following consecutive studies, 19 *Amphisphaeria* species have been accepted [4,5,6,7,8,9].

*Lepteutypa* was introduced by Petrak [10] with its type *L. fuckelii* (≡ *Massaria fuckelii*) which was collected from Germany. *Lepteutypa* is characterized by scattered, weakly developed eutypoid stroma with a single opening; immersed to semi-immersed, single or clustered perithecia, papillate or short conical ostioles; a cellular peridium; broad, easily broken and numerous paraphyses; 8-spored, cylindrical asci; and oblong, multicellular, pigmented ascospores with thick epispore [10]. Fifteen *Lepteutypa* species have been published based on above morphology or morpho-phylogenetic studies [9,11,12,13,14,15,16,17]. Jaklitsch et al. [16] proposed a neotype for *L. fuckelii* on *Tilia cordata* from Belgium, which has aggregated perithecial colonies and 5-septate ascospores. However, in the original generic description, *L. fuckelii* is characterized by eutypoid stroma as a key character in *Lepteutypa* [10]. Nevertheless, the aggregated perithecia in the generic description by Petrak [10] were considered to be an eutypoid stroma [16].

Petrak [18] introduced *Lepteutypella*, which is typified by *L. allospora* (≡ *Cladosphaeria allospora*). The genus has poorly developed, small, eutypoid, spotty stromata, thin, sub-hyaline, fibrous tissue layer around the ostiole as a distinct clypeus, perithecia with a conical ostiole, broad, simple, very delicate paraphyses (metaphyses), 8-spored, delicate, cylindrical asci and broadly ellipsoidal, multicellular, dark coloured, thick episporous ascospores. However, *Lepteutypella* has hitherto been regarded as a synonym of *Lepteutypa* [19].

The presence of multiseptate ascospores in *Lepteutypa* is a key indicator to differentiate *Lepteutypa* from *Amphisphaeria* species. However, some *Lepteutypa* species, such as *L. uniseptate,* have 1-septate ascospores, which are morphologically similar to *Amphisphaeria*, but were phylogenetically related to *Lepteutypa* in previous studies [9,16,17]. Thus, the ascospore septation is not a specific characteristic for the generic delimitation of two genera. The correlations between concepts based on morphology vs. phylogeny of *Amphisphaeria* and *Lepteutypa* make taxonomic instability of generic delimitation, which needs further studies.

In this study, we evaluate the morphology and phylogeny of accepted species in *Amphisphaeria* and *Lepteutypa*, and synonymize *Lepteutypa* under *Amphisphaeria*. Furthermore, as part of our continuous studies on inconspicuous xylariaceous taxa from China and Thailand, we introduce three novel taxa which are associated with senescent plant substrates.

V.P. Abreu, A.W.C. Rosado & O.L. Pereira introduced *Trochilispora* in Hyde et al. [20], typified by *T. schefflerae* from Brazil. The ITS-LSU phylogenies in Hyde et al. [20] showed that *T. schefflerae* has an affinity to *Hymenopleella hippophaeicola*. However, *Trochilispora* was introduced in Amphisphaeriaceae with an uncertainly of the phylogenetic position. Here, we reconstruct the phylogeny including selected taxa and provide evidence for the phylogenetic placement of *T. schefflerae*.

## 2. Materials and Methods

### 2.1. Sample Collection, Isolation and Morphological Studies

We have been focusing on collecting microfungi from senescent twigs, branches, and culms in both mono- and dicotyledonous plants with emphasis on xylarialean species. Surveys from 2017−2019 investigated several interesting inconspicuous xylariaceous species from northern Thailand [7,21]. In this study, we collected samples from northern Thailand and Sichuan Province, China. Specimens were placed in paper bags and dried at room temperature. External and internal macro-micro structural observations were made as described in Samarakoon et al. [7].

Single spore isolations were carried out as detailed in Chomnunti et al. [22] and germinating spores were transferred aseptically to potato dextrose agar (PDA). The cultures were incubated at 25–30 °C for 4–6 weeks and colonies observed frequently. The type specimens were deposited in the Mae Fah Luang University Herbarium (MFLU), Chiang Rai, Thailand and the Cryptogamic Herbarium, Kunming Institute of Botany, Academia Sinica (HKAS), Kunming, China. Ex-type living cultures were deposited in the Culture Collection at Mae Fah Luang University (MFLUCC) and International Collection of Microorganisms from Plants (ICMP), New Zealand. Facesoffungi and MycoBank numbers are provided as explained in Jayasiri et al. [23] and MycoBank (http://www.MycoBank.org).

### 2.2. DNA Extraction, PCR Amplification and Sequencing

Fresh mycelia were scraped from two-week-old cultures on PDA using sterilized scalpels. Cleaned ascomata were picked up from specimens using sterilized needles. Genomic DNA was extracted using the Ezup DNA Extraction Kit (Sangon Biotech, Shanghai, China) according to the manufacturer’s protocol. The internal transcribed spacer (ITS) and partial 28S large subunit rDNA (LSU) nuclear ribosomal DNA were amplified using ITS5/ITS4 [24] and LR0R/LR5 [25] primers respectively following 94 °C/30 s, 55 °C/50 s, 72 °C/60 s protocol. Partial RNA polymerase II second largest subunit (RPB2) and β-tubulin (TUB2) were amplified using fRPB2-5f/fRPB2-7cR [26] and T1/T22 [27] primers following 95 °C/45 s, 52 °C/50 s, 72 °C/60 s and 95 °C/60 s, 54.5 °C/50 s, 72 °C/90 s protocols, respectively. All the PCR protocols were followed by 35 cycles including 94 °C/5 min initial denaturation and 72 °C/10 min final extension.

The 25 μL total volume of PCR mixture contained 9.5 μL of ddH_2_O, 12.5 μL of 2X PCR Master Mix (TIANGEN Co., China), 1 μL of DNA template, and 1 μL of forward and reverse primers (10 μM each) in each reaction. PCR amplified products were checked on 1% agarose electrophoresis gels stained with GoldView I nuclear staining dye (1 μL/10 mL of agarose). Purification and sequencing of PCR products were done by Invitrogen Biotechnology Co. Ltd., Beijing, China. A consensus sequence for each gene region was assembled in SeqMan (DNAStar, Inc., Madison, WI, USA).

### 2.3. Phylogenetic Analyses

Generated ITS, LSU, RPB2, and TUB2 sequences were subjected to BLASTn searches (https://blast.ncbi.nlm.nih.gov) and related sequences were downloaded from GenBank^®^ (Table 1). The individual gene matrix was aligned using MAFFT v7 (https://mafft.cbrc.jp/alignment/server/large.html; [28]) with E-INS-i and L-INS-i Iterative refinement methods, respectively, and improved when necessary in BioEdit v. 7.0 [29]. All absent sequences were coded as missing data and characters were assessed to be unordered and equally weighted.

Evolutionary models were estimated by using MrModeltest 2.2 [30] and model parameters were selected independently for different gene regions under the Akaike information criterion (AIC) implemented in PAUP v. 4.0b10. The GTR+G+I model was the best-fit model for all loci.

Single (LSU, ITS, RPB2, TUB2) and combined (LSU-ITS, LSU-ITS-RPB2-TUB2) matrices were used for phylogenetic analyses in order to compare the topology with previous studies which only used LSU-ITS matrices. Maximum parsimony (MP) analyses were carried out using PAUP v.4.0b 10 [31]. The parameters were set up with tree bisection-reconnection (TBR). Trees were inferred using the heuristic search option with 1000 random sequence additions, with maxtrees set at 1000. Tree length (TL), consistency index (CI), retention index (RI), relative consistency index (RC), and homoplasy index (HI) were calculated for trees generated under different optimality criteria. The Kishino-Hasegawa tests [32] were performed to determine whether trees were significantly different.

Maximum likelihood (ML) analyses were performed at the CIPRES webportal [33] using RAxML-HPC2 on XSEDE (v 8.2.8) with GTR+G+I model and default parameters, and bootstrapping with 1000 replicates [34].

The Bayesian inference (BI) analysis was generated by using Markov Chain Monte Carlo sampling in MrBayes v3.1.2 [35,36] for 3,000,000 generations using four chains with 100 sample frequencies which products 30,000 trees. The first 3000 (10% from total) trees were the burn-in phase and were discarded. The remaining 27,000 trees were used to calculate the posterior probability (PP). The final alignment and tree were registered in TreeBASE under the submission ID: 26768 (http://www.treebase.org). The resulting trees were viewed in FigTree v.1.4.0 [37] and the final layout was done with Adobe Illustrator^®^ CS5 (Adobe Systems, San Jose, CA, USA).

### 2.4. Cluster Analysis

A cluster analysis was performed to assess morphological similarities of *Amphisphaeria* and *Lepteutypa* taxa. The presence of poorly- (0) or well- (1) developed clypeus; solitary (0) or solitary/aggregated (0.5) or aggregated (1) nature of ascomata; l/w ratio of asci ≤15 (0) or >15 (1); J− (0) or J+ (1) apical ring; l/w ratio of ascospores ≤2.8 (0) or >2.8 (1); one septaum (0) or more than one septum (1) in ascospores; and the absence (0) or presence (1) of a mucilaginous sheath around ascospores of *Amphisphaeria* and *Lepteutypa* species were used for the analysis.

The analysis was conducted using Python 3.8 coupled with SciPy 1.5.1 package. We obtained a dendrogram using the unweighted pair group method with arithmetic mean (UPGMA) algorithm and also obtained the Average distance measure. The dendrogram of obtained results was plotted using Matplotlib 3.3.0 package. The heatmap was obtained in a similar way. Plotting was performed by using Seaborn 0.10.1 (https://docs.scipy.org).

## 3. Results

### 3.1. Topology of Phylogenetic Analyses

The LSU-ITS alignment comprised 1374 characters (LSU 1–786, ITS 787–1374) while the LSU-ITS-RPB2-TUB2 combined alignment comprised 2515 characters (LSU 1–786, ITS 787–1377, RPB2 1378–2212, TUB2 2213–2515) including 56 strains.

**Figure 1:**
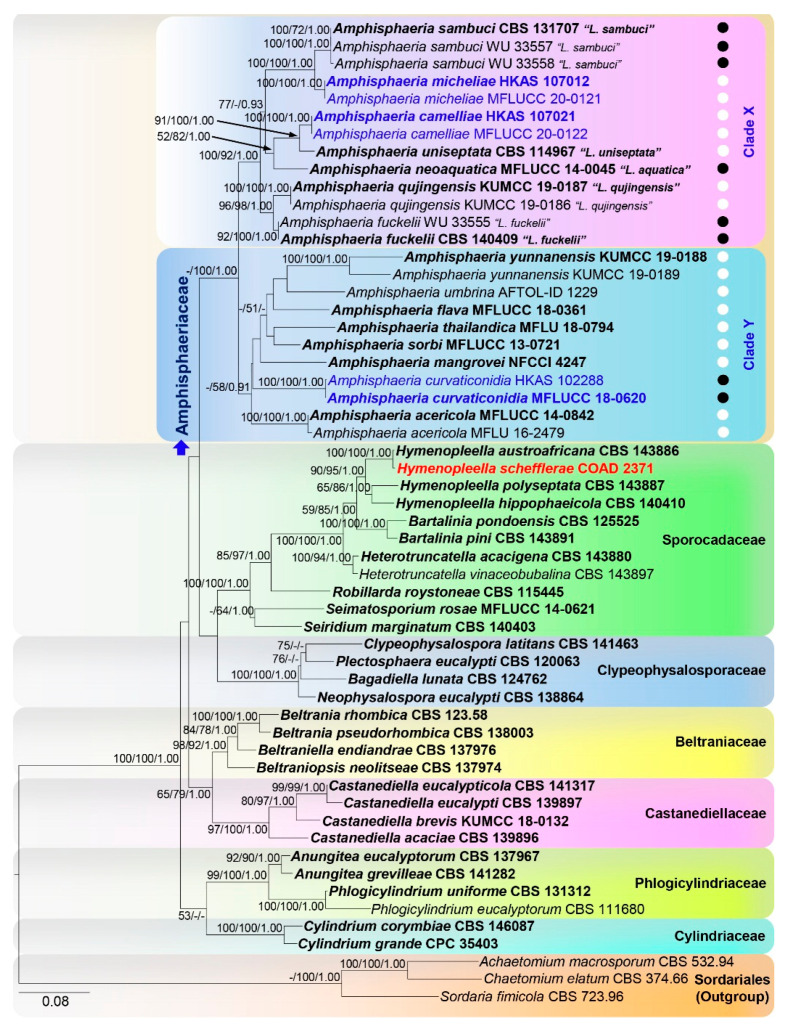
ML tree revealed by RAxML from an analysis of the LSU-ITS-RPB2-TUB2 matrix of the Amphisphaeriaceae and selected families of Amphisphaeriales. Bootstrap supports (≥50%) of MP and ML and the posterior probability values (≥0.9) of BI analyses are indicated above or below the respective branches. Newly generated sequences are in blue and type strains are in bold. The tree is rooted to *Achaetomium macrosporum*, *Chaetomium elatum,* and *Sordaria fimicola* (Sordariales). The scale bar represents the expected number of nucleotide substitutions per site. Ascospore septation among taxa are shown in white (one septum) and black (more than one septum) circles. Taxon in red denotes topological conflict with previous phylogenies. Previous taxa are in “•”.

We follow LSU-ITS-RPB2-TUB2 to show the relationship among related taxa here (Figure 1). The best scoring RAxML tree with a final likelihood value of—1224.694904 is presented. The matrix had 1227 distinct alignment patterns, with 28.04% of undetermined characters or gaps. Estimated base frequencies were as follows; A = 0.247366, C = 0.234669, G = 0.265996, T = 0.251970; substitution rates AC = 1.117478, AG = 3.507552, AT = 1.477282, CG = 1.121674, CT = 6.681156, GT = 1.000000; gamma distribution shape parameter α = 0.485915. The ML, MP, and BY phylogenetic trees resulting from analyses of the alignment of LSU-ITS were different among taxa within the Amphisphaeriaceae (Figure S1). ML and BY results using LSU-ITS-RPB2-TUB2 combined matrix were similar in topologies, and Amphisphaeriaceae was comprised of two well-supported clades containing *Amphisphaeria* species (clade Y) and species that had previously been classified under the genus *Lepteutypa* (clade X). *Amphisphaeria curvaticonidia* (HKAS 102288, MFLUCC 18-0620) formed a basal lineage to all the other amphisphaeriaceous taxa in only MP analysis by using the LSU-ITS-RPB2-TUB2 combined matrix.

The maximum parsimony dataset consisted of 2515 characters, of which 1430 were constant, 881 parsimony-informative (35.03%), and 204 parsimony-uninformative. The parsimony analysis of the data matrix resulted in 1000 equally most parsimonious trees with a length of 3898 steps (CI = 0.445, RI = 0.671, RC = 0.299, HI = 0.555) in the first tree.

*Amphisphaeria curvaticonidia* (HKAS 102288, MFLUCC 18-0620; 100%/100%/1.00 PP) formed a clade with poor statistical support. *Amphisphaeria camelliae* (HKAS 107021, MFLUCC 20-0122; 100%/100%/1.00 PP) is sister to “*Lepteutypa uniseptata*” CBS 114967 (91%/100%/1.00 PP). *Amphisphaeria micheliae* (HKAS 107012, MFLUCC 20-0121; 100%/100%/1.00 PP) formed a clade sister to “*L. sambuci*” (CBS 131707, WU 33557 and WU 33558) with strong statistical support (100%/100%/1.00 PP). The strain “*Trochilispora schefflerae*” (COAD 2371) clustered with *Hymenopleella austroafricana* (CBS 143886) with high statistical support (100%/100%/1.00 PP) in Sporocadaceae.

The ITS sequence of “*Lepteutypa uniseptata*”; CBS 114967 (MH553979) is dubious because it appeared highly similar to *Robillarda* species in a BLASTn search and clustered in Sporocadaceae in our ITS-based phylogenies, which is why it has been excluded from our final analyses.

### 3.2. Cluster Analysis

**Figure 2:**
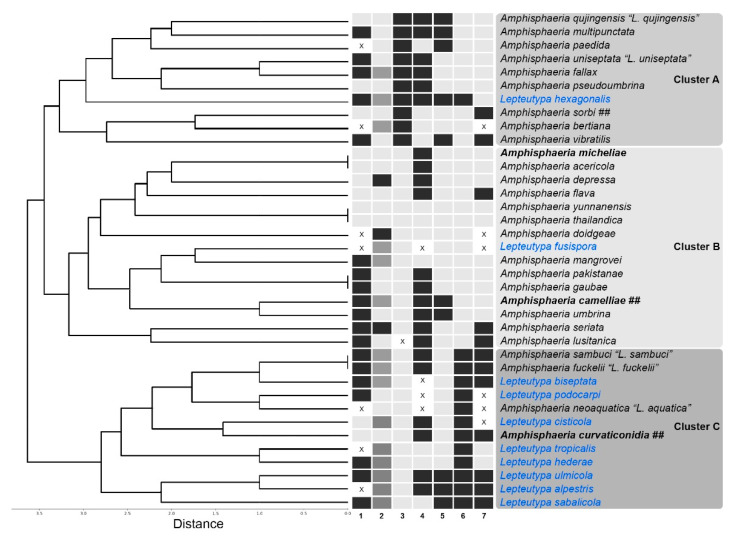
Cluster analysis dendrogram showing similarity among *Amphisphaeria* and *Lepteutypa* species (linkage algorithm paired group UPGMA; Average Distance measure). Characteristics: (1) nature of the clypeus; (2) nature of the ascomata; (3) l/w ratio of asci; (4) apical ring; (5) l/w ratio of ascospores; (6) ascospore septation; and (7) mucilaginous sheath around ascospores. “X” denotes missing data. Newly introduced species are in bold. Taxa which needed to be revised are in blue. Holomorphic taxa are denoted as “##”.

The cluster analysis was comprised of 37 species with seven characters (Figure 2). There are three clusters (A, B and C). The type of species of *Amphisphaeria*; *A. umbrina* and the type of “*Lepteutypa*”; “*L. fuckelii*” cluster with clusters B and C, respectively. Species in cluster A shared asci l/w greater than 15. Clusters B and C were mainly distinguished by having 1-septate (B) and multiseptate (C) ascospores. All other characteristics were distributed among the species without a unique characteristic for each genus.

### 3.3. Taxonomy

#### 3.3.1. Amphisphaeria

*Amphisphaeria* Ces. & De Not., Comm. Soc. crittog. Ital. 1(fasc. 4): 223 (1863), emend.

MycoBank: MB173; Facesoffungi number: FoF02099

= *Poikiloderma* Füisting, Bot. Ztg. 26: 369 (1868)

= *Massariopsis* Niessl, Verh. nat. Ver. Brünn 14: 199 (1876)

= *Phorcys* Niessl, Verh. nat. Ver. Brünn 14: 200 (1876)

= *Conisphaeria* Cooke, Grevillea 7(no. 43): 86 (1879)

= *Massariella* Speg., Anal. Soc. cient. argent. 9(4): in tabula [facing p. 192) (1880)

= *Lepteutypa* Petr., Annls mycol. 21(3/4): 276 (1923)

= *Rhynchostomopsis* Petr. & Syd., Annls mycol. 21(5/6): 377 (1923)

= *Lepteutypella* Petr., Annls mycol. 23(1/2): 98 (1925)

= *Macrothelia* M. Choisy, Bull. mens. Soc. linn. Soc. Bot. Lyon 18: 107 (1949)

Saprobic on woody branches, twigs and culms in terrestrial, freshwater, and mangrove habitats. Sexual morph: appearing as slightly raised, black dots on host surface, often surrounded by a dark or light-coloured, halo-like area. *Pseudostromata,* when present, are made up of host cells and brown to black fungal hyphae. The *Clypeus* is often poorly developed. *Ascomata:* perithecial, scattered, solitary or clustered, immersed, erumpent or rarely superficial, globose, subglobose or ellipsoidal, coriaceous, dark brown, papillate ostiole. *Papilla:* narrow, conical, periphysate, often umbilicate. *Periphyses:* hyaline, filamentous. *Peridium:* two-layered, with an outer layer comprising dark brown cells arranged in a *textura angularis* and inner layer comprising thin-walled, hyaline cells. *Hamathecium:* comprising numerous, filamentous, septate, slightly tapering paraphyses. *Asci:* 8-spored, unitunicate, cylindrical, indistinctly pedicellate, apex rounded, with J+ or J− apical ring. *Ascospores:* uniseriate to overlapping uniseriate, light brown to dark brown, ellipsoid to fusiform, rarely curved, 1–3-septate, not or slightly constricted at the septum, rarely 2–4(6)-distoseptate, smooth-walled, some guttulate, with or without a mucilaginous sheath. Asexual morph: Coelomycetous. *Conidiomata:* solitary or aggregated, globose to sub-globose, dark brown. *Peridium:* comprised of thick-walled, septate, brown mycelium. *Conidiophores:* septate, branched, thick-walled, hyaline to light brown. *Conidiogenous cells:* elongated, thin-walled, septate, hyaline, annellidic. *Conidia:* hyaline, elongate-fusiform, 1-celled with or without appendage derived from the middle of the conidia, smooth-walled.

Type species: *Amphisphaeria umbrina* (Fr.) De Not., Sfer. Ital.: 69 (1863)

MycoBank: MB222981; Facesoffungi number: FoF08735

≡ *Sphaeria umbrina* Fr., Syst. mycol. (Lundae) 2(2): 461 (1823) nom. sanct.

= *Sphaeria mammillaris* Schumach., Enum. pl. (Kjbenhavn) 2: 157 (1803)

= *Sphaeropsis conica* Lév., in Demidov, Voyage dans la Russie Meridionale et la Crimeé, par la Hongrie, la Valachie et la Moldavie 2: 112 (1842)

= *Diplodia conica* (Lév.) Lév., Annls Sci. Nat., Bot., sér. 3 9: 258 (1848)

= *Amphisphaeria conica* (Lév.) Ces. & De Not., Comm. Soc. crittog. Ital. 1(fasc. 4): 224 (1863)

= *Hypocrea gelatinosa* var. *umbrina* (Fr.) Sacc., Syll. fung. (Abellini) 2: 524 (1883)

= *Kirschsteiniella conica* (Lév.) Petr., Sydowia 7(1-4): 57 (1953)

Typus: Italy, Flaventino, on trunk of *Ulmus* sp. (Ulmaceae), Nov. 1860, L. Caldesi, Rabenhorst Fungi Europaei 327 (RO, epitype).

Notes: Fries [57] described *Sphaeria umbrina* on the wood of *Alnus* (Betulaceae) from Sweden. Cesati and de Notaris [1] introduced *Amphisphaeria* without a type species. Petrak [10] revisited the morphology of the *Amphisphaeria umbrina* on the bark of *Ulmus* (Ulmaceae) from Italy and proposed it as the type of species of *Amphisphaeria*. Consecutive studies from Clements and Shear [58], Müller and Arx [59], and Korf [60] accepted *A. umbrina* as the type species of *Amphisphaeria* represented by *Sphaeria umbrina*. Hyde et al. [61] re-examined a specimen of *S. umbrina* from the collection of Cesati and de Notaris [1], also which was used by Petrak [10] for description. The specimen was proposed as the epitype for *A. umbrina*. Hyde et al. [61] provided another *A. umbrina* specimen from Austria on *Quercus petraea* (Fagaceae) (AFTOL-ID 1229; CBS 172.96). *Amphisphaeria umbrina* is distributed in temperate habitats on *Quercus* (Fagaceae), *Salix* (Salicaceae), *Tilia* (Malvaceae) and *Ulmus* (Ulmaceae) [61]. In addition, *Amphisphaeria* has been described from *Alnus* (Betulaceae), *Fraxinus* (Oleaceae), *Quercus* (Fagaceae), *Salix* (Salicaceae) and *Ulmus* (Ulmaceae) [62].

Petrak [10] introduced *Lepteutypa* to accommodate species with eutypoid stroma and multicellular ascospores different from *Amphisphaeria*. *Lepteutypa* species do not possess eutypoid stroma in their morphological descriptions, figures, and illustrations except aggregated ascomata. Jaklitsch et al. [16] proposed a neotype for *Lepteutypa fuckelii*, the type species of the genus, which has scattered or aggregated ascomata on the host but not eutypoid stroma. Jaklitsch et al. [16] further noted that Petrak [10] interpreted those aggregated perithecial colonies as eutypoid stromata. Among previously known *Lepteutypa* species, *L. alpestris*, *L. cisticola*, *L. fusispora*, *L. hederae* and *L. tropicalis* also possess aggregated perithecia, while *L. hexagonalis* possesses two perithecia under a single clypeus rarely. The aggregated ascomata also can be observed in *A. bertiana* and *A. seriata*.

*Amphisphaeria* and *Lepteutypa* were previously separated based on stromatic nature and ascospore septation. However, with the introduction of *Amphisphaeria camelliae* and *A. micheliae*, which are typical of *Amphisphaeria* and not “*Lepteutypa”* where they cluster, we conclude the *Amphisphaeria* and “*Lepteutypa”* are congeneric. There is no distinct characteristic used to separate *Amphisphaeria* (clade Y) and “*Lepteutypa*” (clade X) clusters in Figure 1. Both clusters have 2-celled and multicelled ascospores. No single characteristic exists in one clade. The predicament here is whether to follow the molecular data, which indicates there are two distinct genera (*Amphisphaeria* and *Lepteutypa*) or the morphological data which indicates the group is one genus (*Amphisphaeria*). We adopt a single genus, *Amphisphaeria*, due to the morphological similarity. However, further studies with fresh collections may resolve the taxonomic relationships in Amphisphaeriaceae and sexual asexual connections.

The coelomycetous asexual morph of *Amphisphaeria sorbi* is the only asexual record in Amphisphaeriaceae. Several morphological records of pestaloid-like asexual morphs for “*Lepteutypa”* have been suggested, but not confirmed [63]. In our study, we obtained two asexual morphs which are not pestaloid-like, but similar to the coelomycetous asexual morph of *A sorbi*. Since there is lack of sequence data for most of the “*Lepteutypa”* species, here we provided five taxonomic combinations of “*Lepteutypa”* species which have molecular data. However, the remaining “*Lepteutypa”* species are treated as ambiguous taxa and need to be revisited with molecular and morphology data in further studies.

#### 3.3.2. Additional Accepted Species in Amphisphaeria

*Amphisphaeria acericola* Senan., Camporesi & K.D. Hyde, Phytotaxa 403(4): 285–292 (2019)

MycoBank: MB553774; Facesoffungi number: FoF03594

Typus: Italy, Province of Forlì-Cesena, Galeata, Strada San Zeno, on a branch of *Acer campestre* (Sapindaceae), 26 Mar 2014, E. Camporesi, IT 1779 (MFLU 16-2479, holotype); ex-type living culture MFLUCC 14-0842.

Notes: *Amphisphaeria acericola* is similar to *A. pseudoumbrina* in having immersed, oblate ascomata, J+, a discoid apical ring, cylindrical asci with rugose-walled, brown, uniseptate ascospores, but different in having non-clypeate ascomata, peridium with *textura angularis* cells, and aseptate, cellular paraphyses. The LSU-ITS phylogeny of *A. acericola* forms a basal clade distinct from other *Amphisphaeria* species.

*Amphisphaeria bertiana* Fairm., Proc. Rochester Acad. Sci. 4: 217 (1906)

MycoBank: MB190709; Facesoffungi number: FoF08738

Typus: USA, New York, Lyndonville, in cavities at the end of a rotting log, Oct. 1905, Fairman (CUP, holotype).

Notes: Wang et al. [2] re-examined the holotype. *Amphisphaeria bertiana* has erumpent or superficial ascomata on a subiculum and J− apical ring.

**Figure 3:**
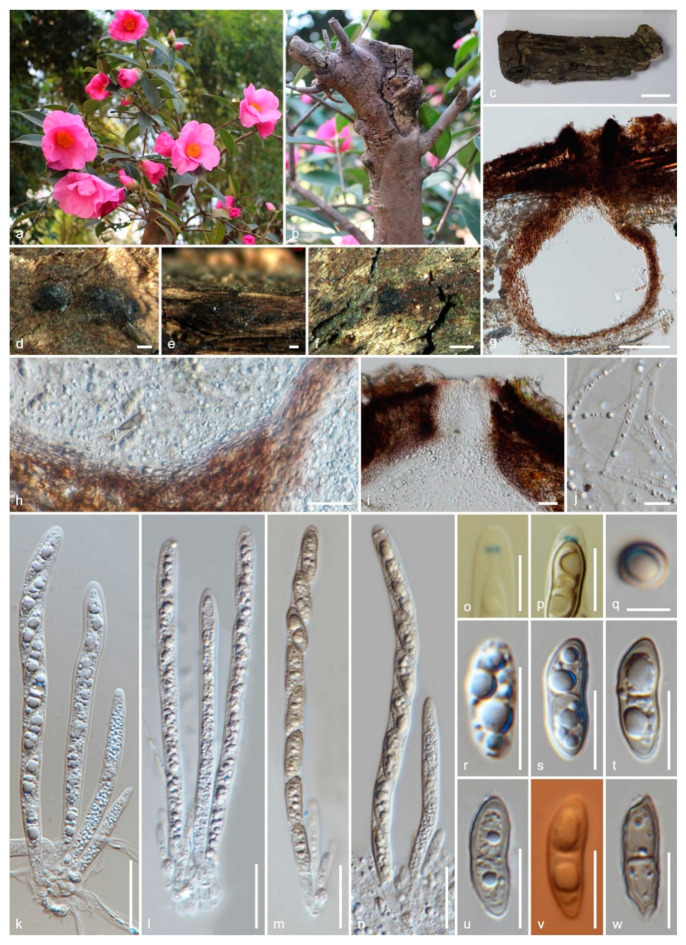
The sexual morph of *Amphisphaeria camelliae* (HKAS 107021, holotype). (**a**,**b**) Host *Camellia japonica*; (**c**) substrate; (**d**–**f**) ascomata on the substrate; (**g**) vertical section of ascoma; (**h**) peridium; (**i**) ostiole; (**j**) paraphyses; (**k**–**n**) asci; (**o**,**p**) apical ring bluing in Melzer’s reagent; (**q**) ascospore top view; (**r**–**w**) ascospores (v in Congo Red). Scale bars are set at (**c**) 1 cm; (**f**) 500 µm; (**d**,**e**) 200 µm; (**g**) 100 µm; (**h**,**i**,**k**–**n**) 20 µm; (**o**,**p**,**r**–**w**) 10 µm; (**q**) 5 µm.

**Figure 4:**
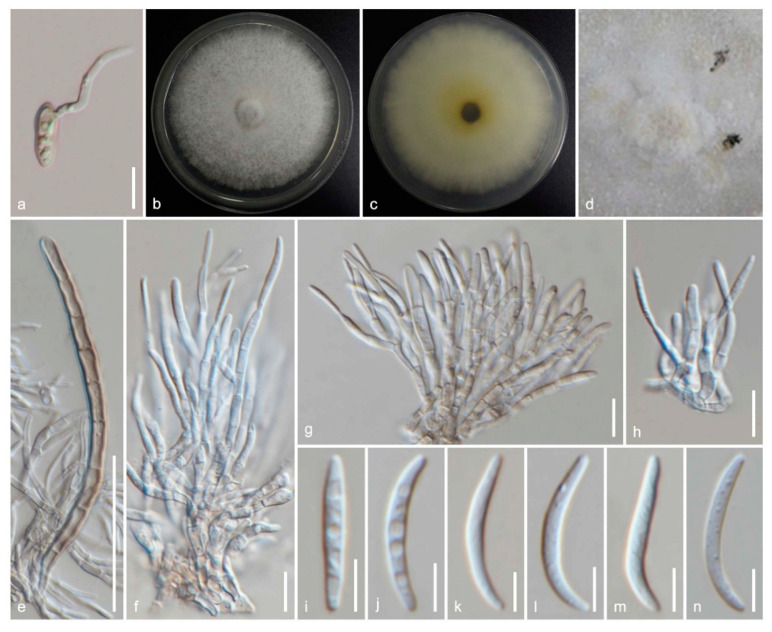
The asexual morph of *Amphisphaeria camelliae* (MFLUCC 20-0122, ex-type living culture). (**a**) Germinating ascospore; (**b**) upper view, (**c**) reverse view of the 2 weeks old colony on PDA; (**d**) conidiomata in the culture; (**e**) setae; (**f**–**h**) conidiophores, conidiogenous cells and conidiogenesis; (**i**–**n**) conidia. Scale bars are set at (**e**) 50 µm; (**a**,**f**–**h**) 10 µm; (**i**–**n**) 5 µm.

*Amphisphaeria camelliae* Samarak., Jian K. Liu & K.D. Hyde, sp. nov. Figure 3 and Figure 4

MycoBank: MB836110; Facesoffungi number: FoF08740

Etymology: The specific epithet reflects the host genus *Camellia*.

Holotype: HKAS 107021

Saprobic on the dead wood of *Camellia japonica* (Theaceae). **Sexual morph:** *Ascomata:* 300–480 μm high × 160–310 μm diameter (M = 410 × 260 μm, *n* = 5), immersed, visible as black spots covered with pale brown and blackish area, solitary or aggregated, scattered, globose to subglobose, papillate ostiole 80–150 μm high × 50–85 μm diameter (M = 110 × 60 μm, *n* = 5), centric. *Periphyses:* 1–2 μm wide (M = 1.5 μm, *n* = 20), hyaline, short. *Peridium:* two-layered; *outer layer* 5–7 μm (M = 5.5 μm, *n* =10), dense, reddish-brown cells of *textura angularis* 8–15 × 1–2 μm (M = 10.8 × 1.6 μm, *n* = 15), thick-walled. *Inner layer:* 7–12 μm (M = 9.5 μm, *n* =10), loosely arranged, hyaline cells of *textura angularis* 9.5–18 × 1–3 μm (M = 12 × 1.5 μm, *n* = 15), thin-walled, loosely arranged. *Paraphyses:* 2–4 μm wide (M = 3 μm, *n* = 20), hyaline, highly delicate, cellular, constricted septate, guttulate; 1–2 μm wide (M = 1.5 μm, *n* = 20), hyaline, filiform, longer than asci, blunt end, cellular, guttulate, embedded in a gelatinous matrix. *Asci:* 85–130 × 5–8 μm (M = 110 × 6.5 μm, *n* = 25), 8-spored, unitunicate, cylindrical, thin-walled, short-pedunculate, apically rounded, with a J+, discoid apical ring. *Ascospores:* 12–17.5 × 4–5.5 μm (M = 15 × 5 μm, *n* = 40), l/w 3.1, uniseriate, oblong or narrowly fusiform, first hyaline, guttulate, turning yellow to yellowish-brown, with a median septum, slightly constricted at the septum, straight to slightly curved, smooth-walled. **Asexual morph:** Coelomycetous. *Conidiomata:* superficial on PDA, solitary or aggregated, globose, dark brown. *Conidiophores:* 24–40 × 1–3 μm (M = 31 × 2 μm, *n* = 15), arising from peridium, septate, branched, thick-walled, light brown to hyaline. *Setae:* 60–92 × 3.5–5 μm (M = 76 × 4.5 μm, *n* = 5), septate, thick-walled, blunt end, brown to light brown. *Conidiogenous cells:* 7.5–14.5 × 1.5–2.5 μm (M = 11.5 × 2 μm, *n* = 15), elongated conical, thin-walled, hyaline, annellidic, guttulate. *Conidia:* 14.5–18 × 1.5–2.5 μm (M = 16 × 2 μm, *n* = 25), elongate-fusiform, curved, smooth-walled, hyaline, guttulate.

Culture characteristics: colonies on PDA, reaching 16–17 mm diameter after one week at 25 °C, the colonies are flat, circular, dense, with a smooth surface, entire margin, and white to light brown. Media become pale brown; reverse light brown at center and dirty white edges.

Material examined: China, Sichuan Province, Chengdu, University of Electronic Science and Technology of China (UESTC) campus, on the wood of *Camellia japonica* (Theaceae), 30 September 2019, M.C. Samarakoon, SAMC254 (HKAS 107021, holotype; MFLU 20-0504, isotype); ex-type living culture MFLUCC 20-0122.

Notes: Our specimens have solitary and aggregated, immersed ascomata with two-layered peridium, unitunicate asci with J+, a discoid apical ring, and brown ascospores similar to amphisphaeriaceous species. *Amphisphaeria camelliae* possesses 1-septate ascospores similar to *A. uniseptata*, but differs in having single or aggregated, globose to subglobose (vs. single, subglobose or applanate) ascomata, thin paraphyses (3.1 vs. 5 μm) and large ascospores (l/w 3.1 vs. l/w 2.7). In the phylogenetic analyses, our collection also clusters with *A. uniseptata*. Based on morphology and phylogeny, our collection is introduced as a novel species *A. camelliae*.

**Figure 5:**
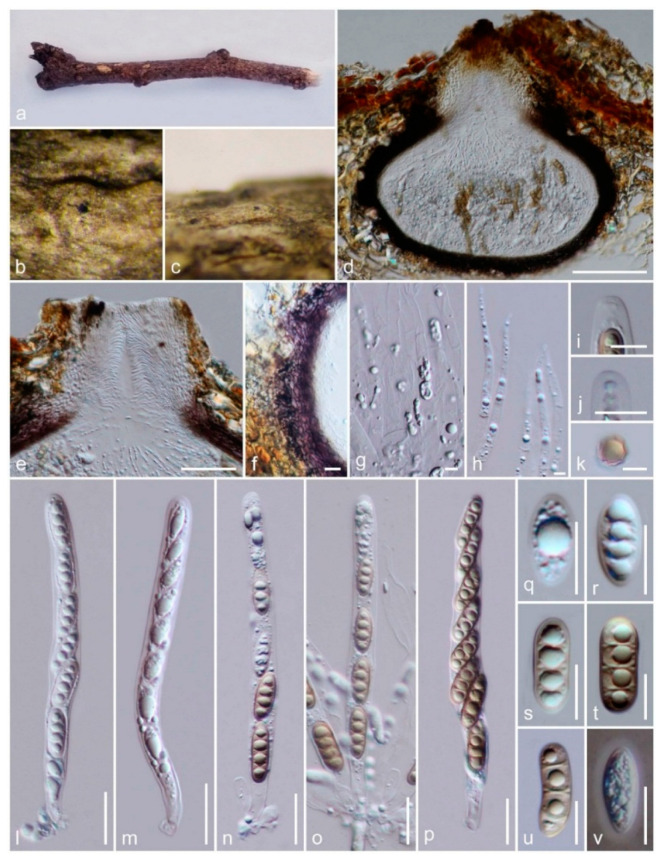
Sexual morph of *Amphisphaeria curvaticonidia* (MFLU18-0789, holotype). (**a**–**c**) Ascomata on the substrate; (**d**) vertical section of ascoma; (**e**) ostiole; (**f**) peridium; (**g**,**h**) paraphyses; (**i**) apical ring (in water); (**j**) apical ring bluing in Melzer’s reagent; (**k**) ascospore (top view); (**l**–**p**) asci; (**q**–**v**) ascospores (v in Indian Ink). Scale bars are set at, (**d**) 100 µm; (**e**) 50 µm; (**f**,**l**–**p**) 20 µm; (**i**,**j**,**q**–**v**) 10 µm; (**g**,**h**,**k**) 5 µm.

**Figure 6:**
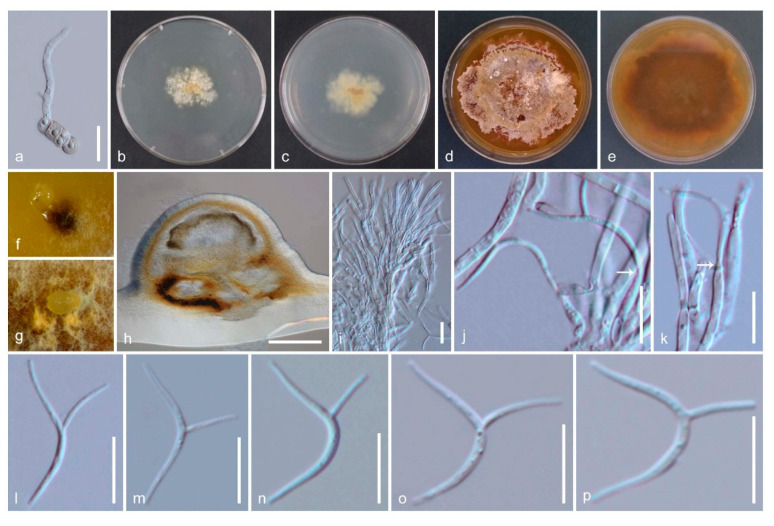
Asexual morph of *Amphisphaeria curvaticonidia* (MFLUCC 18-0620, ex-type living culture). (**a**) Germinating ascospore; (**b**) upper view, (**c**) reverse view of the 2 weeks old colony on PDA; (**d**) upper view, (**e**) reverse view of the 6 weeks old colony on PDA; (**f**,**g**) conidiomata in the culture; (**h**) vertical section of conidiomata in dried PDA; (**i**–**k**) conidiophores, conidiogenous cells, and conidiogenesis (white arrows show conidia attachment); (**l**–**p**) conidia. Scale bars are set at (**h**) 200 µm; (**a**) 20 µm; (**i**–**p**) 10 µm.

*Amphisphaeria curvaticonidia* Samarak., Promp. & K.D. Hyde, sp. nov. Figure 5 and Figure 6

MycoBank: MB836111; Facesoffungi number: FoF08742

Etymology: The specific epithet curvaticonidia reflects the curved conidia.

Holotype: MFLU 18-0789

Saprobic on a dead branch. **Sexual morph:** *Ascomata:* 320–390 μm high × 360 410 μm diameter, (M = 350 × 385 μm, *n* = 8), immersed, visible as black spots, solitary, scattered, globose to subglobose or ovoid, papillate ostiole 75–140 μm wide (M = 110 μm, *n* = 5), yellowish-brown, centric. *Periphyses:* 1.5–3 μm wide (M = 2.5 μm, *n* = 20), hyaline, short. *Peridium:* two-layered; *outer layer:* 18–26 μm (M = 21 μm, *n* =15), reddish-brown cells of *textura angularis* 7–21 × 1.5–4 μm (M = 12 × 2.5 μm, *n* = 20), thick-walled. *Inner layer:* 5.5–11 μm (M = 8 μm, *n* =15), hyaline cells of *textura angularis* 8–20 × 1–2.5 μm (M = 12 × 2 μm, *n* = 20), thin-walled, loosely arranged. *Paraphyses:* 3.5–5 μm wide (M = 4.5 μm, *n* = 20), hyaline, highly delicate, cellular, constricted septate, guttulate; 2–3.5 μm wide (M = 3 μm, *n* = 20), hyaline, filiform, longer than asci, blunt end, cellular, guttulate, embedded in a gelatinous matrix. *Asci:* 121–162 × 10.5–17.5 μm (M = 135 × 12.5 μm, *n* = 25), 8-spored, unitunicate, cylindrical, thin-walled, short-pedunculate, apically rounded, with a J+, discoid apical ring. *Ascospores:* 17–23 × 6–9 μm (M = 20.5 × 7.5 μm, *n* = 50), l/w 2.7, uniseriate, oblong or narrowly fusiform, first hyaline with a thin mucous sheath, turning yellow to yellow-brown, with 1 median, slightly constricted euseptum and two distosepta, straight to slightly curved, smooth-walled, with 8 longitudinal ridges which render the ascospore to appear octagonal in transverse section. **Asexual morph:** Coelomycetous. *Conidiomata:* 590–640 μm high × 370–490 μm diam. (M = 615 × 425 μm, *n* = 3), immersed in PDA, solitary or aggregated, globose to sub-globose, dark brown. *Peridium:* comprised of thick-walled, septate, brown mycelium. *Conidiophores:* 11–17 × 1.5–2.5 μm (M = 14 × 2 μm, *n* = 15), septate, branched, thick-walled, hyaline to light brown. *Conidiogenous cells:* 7–17 × 2–2.5 μm (M = 12.5 × 2.2 μm, *n* = 15), elongate, thin-walled, hyaline, annellidic, guttulate. *Conidia:* 23.5–30 × 1–2 μm (M = 26.5 × 1.5 μm, *n* = 25), hyaline, cylindrical, curved, smooth-walled, appendage derived from the middle of the conidia cell, 6–10.5 μm long (M = 8.5 μm, *n* = 20), curved.

Culture characteristics: Colonies on PDA, reaching 9–10 mm diameter after 2 weeks at 25 °C; colonies are flat, irregular, and dense, with immersed and superficial mycelia, with a rough surface, fimbriate margin, white to light brown becoming orange to dark brown, media becoming brown; reverse light brown at center with dirty white edges, later becoming yellowish-brown.

Material examined: Thailand, Chiang Rai, Thoeng, Ban Mae Loi Rai, on a dead branch, 19°54′ N 100°06′ E, 350 m msl, 11 September 2017, M.C. Samarakoon, SAMC040 (MFLU 18-0789, holotype; HKAS 102288, isotype); ex-type living culture MFLUCC 18-0620.

Notes: Our collection shares immersed, clypeate, globose to subglobose ascomata with inner hyaline and outer brown peridium, sepatate, flexuose paraphyses and ellipsoidal, brown ascospores typical for *Amphisphaeria*. The J+ discoid apical ring is similar to several other species, including *A. flava* described from Thailand. However, *A. flava* differs from our new collection in having a halo on the host surface around the ostiole and 1-septate ascospores. *Amphisphaeria mangrovei* differs from our strains with J− and an apical ring in asci. In addition, some of the immature ascospores are covered with a thin mucilaginous sheath as in *A. flava*, *A. lusitanica*, *A. seriata*, *A. sorbi* and *A. vibratilis*. In addition, our collection has 1 median, slightly constricted at the euseptum, and two distosepta which are characteristic to *A. depressa*. Wang et al. [2] re-examined the herbarium specimen of *A. depressa* and noted that this unusual distoseptate ascospores characteristic is not matched with the generic description. However, based on unituniate asci with J+, apical ring, Wang et al. [2] accepted this species in Amphisphaeriaceae. The asexual coelomycetous from the culture of our strain is similar to the only known asexual morph of *A. sorbi* by having septate, branched, hyaline conidiophores, elongated conical, thin-walled, septate, hyaline, annellidic conidiogenous cells, and elongate-fusiform, 1-celled, hyaline conidia. The asexual morph of our strain is characterized with curved appendage derived from the middle of the conidia cell. Here we introduce our new collection as *A. curvaticonidia*.

*Amphisphaeria depressa* Petr., Sydowia 7(5–6): 381 (1953)

MycoBank: MB292502; Facesoffungi number: FoF08743

Typus: USA, Hawaii, Kaihea, Oahu, on *Cassia bicapsularis* (Fabaceae), 24 Feb. 1928, Shear (W 11997, holotype; BPI 618577, isotype).

Notes: Wang et al. [2] re-examined the holotype and remarked the distoseptate ascospores are an unusual character of this species and accommodated in *Amphisphaeria* until further collections are made.

*Amphisphaeria doidgeae* Marinc., M.J. Wingf. & Crous, in Marincowitz et al., CBS Diversity Ser. (Utrecht) 7: 20 (2008)

MycoBank: MB506197; Facesoffungi number: FoF08744

Typus: South Africa, Western Cape Province, Kleinmond, on twig litter of *Leucadendron* sp. (Proteaceae), 11 July 2000, S. Marincowitz, S.L. 485 (PREM 59556, holotype).

Notes: *Amphisphaeria doidgeae* is characterized by having broader peridium (59–72 µm), septate and branched paraphyses, asci with a J– apical ring and broadly fusiform with a base often projected and truncate ascospores.

*Amphisphaeria fallax* De Not., Hedwigia 4: 21 (1865)

MycoBank: MB187770; Facesoffungi number: FoF08745

Typus: Czech Republic, Weiβkirchen, on *Quercus robur* (Fagaceae), Apr. 1936, F. Petrak (DAOM 148328, neotype).

Notes: Wang et al. [2] designated a neotype by observing a slide collection from *Quercus robur* deposited by Petrak in 1936 with similar morphology.

*Amphisphaeria flava* Samarak. & K.D. Hyde, Phytotaxa 391 (3): 210–211 (2019)

MycoBank: MB555396; Facesoffungi number: FoF04976

Typus: Thailand, Chiang Mai, Chang Wat, Amphoe Mae Taeng, Tambon Pa Pae, on a senescent branch, 01 September 2017, M.C. Samarakoon, SAMC019 (MFLU 18-0102, holotype; HKAS 102301, isotype); ex-type living culture MFLUCC 18-0361.

Notes: Samarakoon et al. [7] introduced *A. flava* from Thailand which is easy to observe on the host with its round, pale yellow appearance around the ostiole.

*Amphisphaeria fuckelii* (G.H. Otth) Samarak., Maharachch. & K.D. Hyde, comb. nov.

MycoBank: MB836119; Facesoffungi number: FoF08746

≡ *Massaria fuckelii* G.H. Otth, Mitth. Naturf. Ges. Bern Nr. 654–683: 50 (1868)

= *Massaria fuckelii* Fuckel, Jahrb. Nassauischen Vereins Naturk. 23–24: 155. 1870. [1869–70]

= *Lepteutypa fuckelii* (G.H. Otth) Petr., Ann. Mycol. 21: 276 (1923)

Typus: Belgium, Leuven, Heferlee, Heferleebos, on *Tilia cordata* (Malvaceae), 31 May 2012, P. Bormans (WU 33554, neotype); ex-type living culture CBS 140409.

Notes: Jaklitsch et al. [16] proposed a neotype for *Lepteutypa fuckelii* on *Tilia cordata* from Belgium (see the generic amendment above).

*Amphisphaeria gaubae* (Petr.) Y. Z. Wang, Aptroot & K.D. Hyde, Fungal Diversity Res. Ser. 13: 13 (2004)

MycoBank: MB373366; Facesoffungi number: FoF08748

≡ *Didymosphaeria gaubae* Petr., Sydowia 8: 195. (1954)

Typus: Australia, Australian Capital District, Jervis Bay, on dead leaves of *Lambertia formosa* (Proteaceae), 14 July 1950, Gauba (H, holotype).

Notes: Aptroot [64] transferred *Didymosphaeria gaubae* to *Amphisphaeria* and Wang et al. [2] re-examined the holotype and accepted the transfer to *Amphisphaeria* due to the combination of clypeate perithecia, unitunicate asci with J+ apical ring and 1-septate, slightly curved ascospores.

*Amphisphaeria lusitanica* (Niessl) Y. Z. Wang, Aptroot & K.D. Hyde, Fungal Diversity Res. Ser. 13: 16 (2004)

MycoBank: MB373365; Facesoffungi number: FoF08751

≡ *Phorcys lusitanica* Niessl, Inst. Coimbra 31: 15 (1883)

= *Didymosphaeria lusitanica* (Niessl) Berl. & Voglino, in Saccardo, Syll. fung., Addit. I-IV (Abellini): 115 (1886)

= *Microthelia lusitanica* (Niessl) Kuntze, Revis. gen. pl. (Leipzig) 3(3): 498 (1898)

Typus: Portugal, Figueira da Foz, on *Arundo donax* (Poaceae), June 1880, Moller, 986 (M, holotype).

Notes: The wedge-shaped apical ring and ascospores strongly constricted at the septum have a close affinity to *Arecophila* in Cainiaceae [2].

*Amphisphaeria mangrovei* Devadatha & V.V. Sarma, in Phookamsak et al., Fungal Diversity 95: 161 (2019)

MycoBank: MB554279; Facesoffungi number: FoF04273

Typus: India, Tamil Nadu, Tiruvarur, Muthupet mangroves, on intertidal branches and twigs of *Suaeda monoica* (Chenopodiaceae), 29 October 2016, B. Devadatha (AMH-9948 holotype); ex-type living culture NFCCI-4247.

Notes: Phookamsak et al. [6] introduced *Amphisphaeria mangrovei* on intertidal branches and twigs as the first report of *Amphisphaeria* species associated with mangrove habitats. LSU-SSU-ITS phylogenies revealed that *A. mangrovei* is a sister to *A. sorbi*.

**Figure 7:**
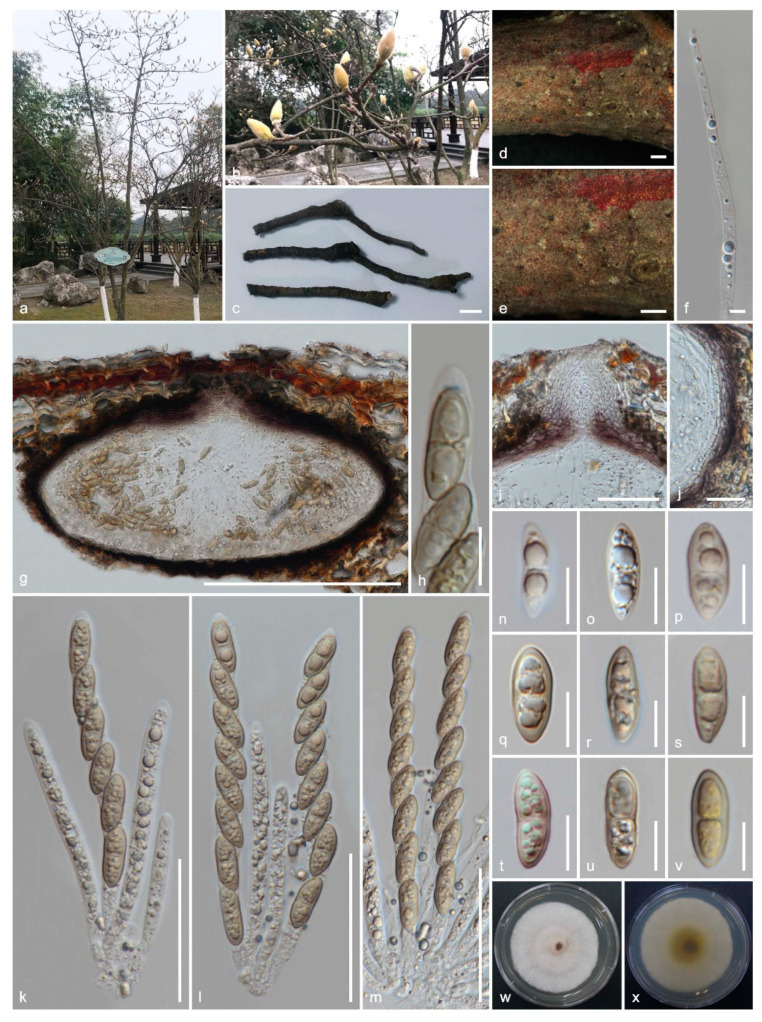
Sexual morph of *Amphisphaeria micheliae* (HKAS 107012, holotype). (**a**,**b**) Host *Michelia alba*; (**c**) substrate; (**d**,**e**) ascomata on the substrate; (**f**) paraphyses; (**g**) vertical section of ascoma; (**h**) apical ring bluing in Melzer’s reagent; (**i**) ostiole; (**j**) peridium; (**k**–**m**) asci; (**n**–**v**) ascospores (v in Melzer’s reagent); (**w**) upper view, (**x**) reverse view of the 2 weeks old colony on PDA. Scale bars are set at (**c**) 1 cm; (**d**,**e**) 500 µm; (**g**) 200 µm; (**i**,**k**–**m**) 50 µm; (**j**) 20 µm; (**h**,**n**–**v**) 10 µm.

*Amphisphaeria micheliae* Samarak., Jian K. Liu & K.D. Hyde, sp. nov. Figure 7

MycoBank: MB836112; Facesoffungi number: FoF08752

Etymology: The specific epithet reflects the host genus *Michelia*.

Holotype: HKAS 107012

Saprobic on a dead branch of *Michelia alba*. Sexual morph: *Ascomata:* 180–210 μm high × 225–370 μm diameter (M = 190 × 300 μm, *n* = 8), immersed, visible as black spots in light coloured area on the host, solitary, scattered, subglobose to oblate, papillate ostiole 60–76 μm high × 34–50 μm diam. (M = 68 × 42 μm, *n* = 8), centric. *Periphyses:* 1–2 μm wide (M = 1.5 μm, *n* = 20), hyaline, short. *Peridium:* two-layered; *outer layer:* 6.5–11.5 μm (M = 8.5 μm, *n* =10), dense, reddish brown cells of *textura angularis* 6.5–12.5 × 1–2.5 μm (M = 9 × 1.5 μm, *n* = 15), thick-walled. *Inner layer:* 4–8 μm (M = 6 μm, *n* =10), loosely arranged, hyaline cells of *textura angularis* 10.5–17.5 × 2.5–4 μm (M = 14.5 × 3 μm, *n* = 15), thin-walled, loosely arranged. *Paraphyses:* 3.5–4.5 μm wide (M = 4 μm, *n* = 20), hyaline, highly delicate, cellular, constricted septate, guttulate, embedded in a gelatinous matrix. *Asci:* 92–135 × 7–10.5 μm (M = 115 × 8.5 μm, *n* = 25), 8-spored, unitunicate, cylindrical, thin-walled, short-pedunculate, apically rounded, with a J+, discoid apical ring. *Ascospores:* 15.5–21 × 6–7.5 μm (M = 18 × 6.5 μm, *n* = 40), l/w 2.7, uniseriate, oblong or narrowly fusiform, first hyaline, guttulate, turning yellow to yellow-brown, 1-septate, slightly constricted at septum, straight to slightly curved, smooth-walled. Asexual morph: Undetermined.

Culture characteristics: colonies on PDA, reaching 20–22 mm diameter after one week at 25 °C; colonies are flat, circular, and dense, with a smooth surface, entire margin, concentrically zonate, white to light brown, media becoming pale brown; reverse yellowish brown at center and light brown edges.

Material examined: China, Sichuan Province, Chengdu, University of Electronic Science and Technology of China (UESTC) campus, on a dead branch of *Michelia alba* (Magnoliaceae), 30 September 2019, M.C. Samarakoon, SAMC244 (HKAS 107012, holotype; MFLU 20-0503, isotype); ex-type living culture MFLUCC 20-0121.

Notes: Two of our collections have solitary, immersed ascomata with two-layered peridium, unitunicate asci with J+, discoid apical ring, and brown ascospores. This collection also has 1-septate ascospores similar to *Amphisphaeria unisepta* and *A. camelliae*. Compared to those two similar species, this collection has subglobose to oblate ascomata and oblong or narrowly fusiform ascospores. The phylogenetic analyses show MFLUCC 20-0121 and HKAS 107012 are closely related to *A. sambuci*, isolated from partly decorticated branches of *Sambucus nigra*. *Amphisphaeria sambuci* is different from our new collection in having large, depressed globose ascomata and oblong-ellipsoid, straight, rarely curved, 2–4(–6)-distoseptate ascospores with a thick mucilaginous sheath. Based on the morphology and phylogeny, here we introduce it as the novel species *A. micheliae*.

*Amphisphaeria multipunctata* (Fuckel) Petr., Annls mycol. 21(3/4): 329 (1923) (as “*Amphisphaeria millepunctata*”)

MycoBank: MB271193; Facesoffungi number: FoF08753

≡ *Diaporthe multipunctata* Fuckel, Jb. nassau. Ver. Naturk. 27–28: 37 (1874) [1873–74]

= *Sphaeria acerina* (Rehm) Cooke & Plowr. (1833)

= *Didymosphaeria acerina* Rehm, Ascomyceten: no. 2237 (1874)

= *Didymosphaeria acerina* var. *fraxini* G. Winter ex Sacc., Syll. fung. (Abellini) 1: 714 (1882)

= *Massariopsis acerina* (Rehm) Kirschst., Annls mycol. 33(3/4): 218 (1935)

Typus: Switzerland, Neuchâtel, Ca. Neuchatel, on dry branches of *Corylus avellana* (Betulaceae), in spring, Morthier, Fuckel, Fungi Rhen. Exs. nr. 2661 (holotype?)

Notes: Aptroot [64] synonymized several *Didymodphaeria* species under *Amphisphaeria multipunctata*. Samuels et al. [65] noted that Petrak [10] has spelled as “*Diaporthe millepunctata*” erroneously and the *Amphisphaeria multipunctata* is the correct name. Aptroot [64] documented the host association of *A. multipunctata* as *Acer campestre* (Aceraceae), *Carpinus betulus*, *Quercus robur* (Fagaceae), *Fraxinus excelsior*, *Syringa vulgaris* (Oleaceae), *Prunus spinosa*, *Sorbus aucuparia* (Rosaceae), *Rhus glabra* (Anacardiaceae) and *Viburnum opulus* (Caprifoliaceae).

*Amphisphaeria neoaquatica* Samarak., Maharachch. & K.D. Hyde, nom. nov.

MycoBank: MB836114; Facesoffungi number: FoF08737

≡ *Lepteutypa aquatica* Z.L. Luo, K.D. Hyde & H.Y. Su, in Luo et al., Fungal Diversity: 99:629 (2019) non-*Amphisphaeria aquatica* (Ellis & Everh.) Berl. & Voglino, in Saccardo, Syll. fung., Addit. I–IV (Abellini): 125 (1886); *Amphisphaeria aquatica* Plöttn. & Kirschst., in Kirschstner, Verh. bot. Ver. Prov. Brandenb. 48: 52 (1906) [1907]

Typus: Thailand, Chiang Rai Province, on submerged decaying wood in a freshwater stream, Nov. 2013, K.D. Hyde, ZL-23 (MFLU 15-0077, holotype); ex-type living culture MFLUCC 14-0045.

Notes: Luo et al. [17] introduced *Amphisphaeria aquatica* (later re-named *A. neoaquatica*) which is similar to *A. uniseptata* by having subglobose, black, immersed ascomata and long cylindrical asci. ITS-LSU phylogeny shows that *A. neoaquatica* is basal to amphisphaeriaceous species [17].

*Amphisphaeria paedida* (Berk. & Broome) Sacc., Syll. fung. (Abellini) 1: 724 (1882)

MycoBank: MB208832; Facesoffungi number: FoF08754

≡ *Sphaeria paedida* Berk. & Broome, Ann. Mag. nat. Hist., Ser. 4 11: 348 (1873)

= *Conisphaeria paedida* (Berk. & Broome) Cooke, in Cooke & Plowright, Grevillea 7(no. 43): 86 (1878)

= *Melanomma paedida* (Berk. & Broome) Cooke (?)

Typus: United Kingdom, on Fagus sylvatica (Fagaceae), Apr. 1859 (K(M), holotype).

Notes: Wang et al. [2] re-examined a specimen of *A. paedida* and noted that the superficial ascomata, J− apical ring and ascospores with thickened septa are characteristic of the species. However, fresh collection and taxonomic revision is needed.

*Amphisphaeria pakistanae* E. Müll. & S. Ahmad, Biologia, Lahore 3: 10 (1957)

MycoBank: MB292509; Facesoffungi number: FoF08755

Typus: Pakistan. Swat, Kalam, on dead branches of *Indigofera* sp. (Fabaceae), 18 Aug. 1952, S. Ahmad (Z+ZT 9032, holotype).

Notes: *Amphisphaeria pakistanae* has large ascomata and relatively small, light brown ascospores [2].

*Amphisphaeria pseudoumbrina* Sacc., Atti Soc. Veneto-Trent. Sci. Nat. 2(1): 112 (1873)

MycoBank: MB225907; Facesoffungi number: FoF08757

Typus: Italy, on the bark of *Acer campestre* (Sapindaceae) (VER, isotype).

Notes: Saccardo [66] described *A. pseudoumbrina* which has oblate ascomata, smaller asci and relatively broader ascospores compared to *A. umbrina*. Wang et al. [2] re-examined the isotype, which was found on *Acer campestre* in Italy, and accepted in *Amphisphaeria*.

*Amphisphaeria qujingensis* (Dissan., J.C. Kang & K.D. Hyde) Samarak., Maharachch. & K.D. Hyde, comb. nov.

MycoBank: MB836127; Facesoffungi number: FoF06506

≡ *Lepteutypa qujingensis* Dissan., J.C. Kang & K.D. Hyde, Phytotaxa 446 (3): 150 (2020)

Typus: China, Yunnan Province, Qujing, on a senescent branch of an unknown host, 06 May 2019, L.S. Dissanayake, DW1137-045 (HMAS 290478, holotype; HKAS 107065, isotype); ex-type living culture KUMCC 19-0187.

Notes: *Amphisphaeria qujingensis* is similar in morphology to *A. fuckelii* in having immersed ascomata, J+ apical ring, and multiguttulate, hyaline to brown ascospores. LSU-ITS phylogenies also showed that *A. qujingensis* is sister to *A. fuckelii* with strong statistical support.

*Amphisphaeria sambuci* (Jaklitsch & Voglmayr) Samarak., Maharachch. & K.D. Hyde, comb. nov.

MycoBank: MB836129; Facesoffungi number: FoF08759

≡ *Lepteutypa sambuci* Jaklitsch & Voglmayr, Persoonia 37: 88 (2016a)

Typus: England, Yorkshire, Worksop, Rotherham, Anston, Anstonstones Wood, on partly decorticated branches of *Sambucus nigra* (Adoxaceae), 16 May 2011, T. Læssøe et al. (WU 33556, holotype); ex-type living culture CBS 131707.

Notes: Jaklitsch et al. [16] introduced *Amphisphaeria sambuci* on mostly decorticated branches of *Sambucus nigra* lying on the ground and sometimes submerged in aquatic habitats from Europe. *Amphisphaeria sambuci* has immersed ascomata, asci with J+, apical ring and ascospores with a central scarcely constricted euseptum and 2–4(–6)-distoseptate.

*Amphisphaeria seriata* M.E. Barr & A.W. Ramaley, Mycotaxon 58: 350 (1996)

MycoBank: MB414608; Facesoffungi number: FoF08760

Typus: USA, Texas, Enchanted rock state park, Gillespie Co., on leaf of *Nolina* sp. (Asparagaceae), 23 October 1993, A.W. Ramaley (BPI 802953, holotype).

Notes: The finely foveolate ascospores are reminiscent of *Arecophila foveata*, but this species differs by having a discoid apical ring [2]. Furthermore, *Arecophila* species are mainly from monocots and *A. seriata* needs to be re-collected and taxonomically revised.

*Amphisphaeria sorbi* Senan. & K.D. Hyde, in Liu et al., Fungal Divers 71: 10 (2015)

MycoBank: MB550904; Facesoffungi number: FoF00414

Typus: Italy, Trento [TN], Dimaro, Folgarida, on the branch of *Sorbus aucuparia* (Rosaceae), 02 August 2013, E. Camporesi, IT 1400 (MFLU 14-0797, holotype); ex-type living culture MFLUCC 13-0721.

Notes: Liu et al. [5] introduced *Amphisphaeria sorbi* with its holomorph and LSU phylogeny from Italy. *Amphisphaeria sorbi* is highly similar to *A. vibratilis* but differs in having small perithecia, wide, non-flexuose paraphyses and smooth-walled ascospores without deeply pigmented septa.

*Amphisphaeria thailandica* Samarak. & K.D. Hyde, Phytotaxa 391 (3): 210–211 (2019)

MycoBank: MB555397; Facesoffungi number: FoF04977

Typus: Thailand, Phayao, Phu Sang, Doi Phu Nang, on a dead branch, 20 July 2017, M.C. Samarakoon, SAMC097 (MFLU 18-0794, holotype; HKAS 102290, isotype).

Notes: *Amphisphaeria thailandica* is distinguished from other *Amphisphaeria* species by having subglobose to oval, hyaline bi-guttulate immature and light brown to greyish bi-guttulate ascospores, lacking a mucilaginous sheath.

*Amphisphaeria uniseptata* (C.K.M. Tsui, K.D. Hyde & Hodgkiss) Samarak., Maharachch. & K.D. Hyde, comb. nov.

MycoBank: MB836132; Facesoffungi number: FoF08763

≡ *Clypeosphaeria uniseptata* C.K.M. Tsui, K.D. Hyde & Hodgkiss, Mycologia 93(5): 1004 (2001)

= *Lepteutypa uniseptata* (C.K.M. Tsui, K.D. Hyde & Hodgkiss) Jaklitsch & Voglmayr, Persoonia 37: 88 (2016a)

Typus: Hong Kong, Tai Po, Lam Tsuen River, on submerged wood, Sept. 1997, R.M. Tsui, EjM 247 (HKU(M) 8095, holotype); ex-type living culture HKUCC 6579.

Notes: Tsui et al. [67] introduced *Amphisphaeria uniseptata* on submerged wood from Hong Kong and noted that it is similar to *A. pakistanae* in having ellipsoidal, brown, 1-sepatate but differs from having thick-walled ascospores. Jaklitsch et al. [16] placed this species in *Lepteutypa* emphasizing the presence of clypeus, long cylindrical asci with the J+ apical ring and uniseriate, ellipsoidal, brown ascospores.

*Amphisphaeria vibratilis* (Fuckel) E. Müll., in Müller & von Arx, Beitr. Kryptfl. Schweiz 11(no. 2): 695 (1962)

MycoBank: MB326176; Facesoffungi number: FoF08764

≡ *Massaria vibratilis* Fuckel, Jb. nassau. Ver. Naturk. 23–24: 154 (1870) [1869–70]

= *Massariella vibratilis* (Fuckel) Sacc., Syll. fung. (Abellini) 1: 716 (1882)

= *Phorcys vibratilis* (Fuckel) J. Schröt., Kryptogamische Flora Schlesiens 3: 381 (1897)

= *Massariella vibratilis* var. *mespili* Pass., in Brunaud, Ann. Soc. Sci. nat. Char.-Marit. 25: 29 (1888)

Typus: Canada, British Columbia, on the stem of *Prunus* sp. (Rosaceae), June 1915, J. Macoun (DAOM, isotype).

Notes: Wang et al. [2] re-examined the isotype. *Amphisphaeria vibratilis* has asci with the J− apical ring and verrucose ascospores with a mucilaginous sheath.

*Amphisphaeria yunnanensis* Dissan., J.C. Kang & K.D. Hyde, in Dissanayake et al. Phytotaxa 446 (3): 144–158 (2020)

MycoBank: MB556876; Facesoffungi number: FoF06505

Typus: China, Yunnan Province, Qujing, on a dead branch of an unknown host, 06 May 2019, L.S. Dissanayake, DW1137-048 (HMAS 290476, holotype; HKAS 107066, isotype); ex-type living culture KUMCC 19-0188.

Notes: *Amphisphaeria yunnanensis* has ascomata with narrow and long ostioles, asci with the J– apical ring and ascospores without a gelatinous sheath.

#### 3.3.3. Taxa Needing Further Revisions

Ten species of *Lepteutypa* lack molecular data and asexual morphs. There are some “*Lepteutypa*” species that have been considered with pestaloid-like asexual morphs as mentioned in the relevant notes below and it is not possible to place them in *Amphisphaeria*. Therefore, these “lepteutypa-like” taxa need to be recollected, and sexual–asexual connections, molecular data, and generic affiliations established. We therefore do not treat them in *Amphisphaeria sensu stricto*.

*Lepteutypa alpestris* (Ellis & Everh.) M.E. Barr, Mycotaxon 46: 56 (1993)

= *Melanomma alpestre* Ellis & Everh., Proc. Acad. nat. Sci. Philad. 46(3): 328 (1894)

Typus: USA, Washington, Mt. Paddo, on dead twigs of *Arctostaphylos nevadensis* (Ericaceae), July 1886, W.N. Suksdorf, 268 (NY, holotype; Washington 342, isotype).

Notes: Ellis and Everhart [68] introduced *Lepteutypa alpestris* with the sexual morph. However, they noted that one perithecium contained stromatic nature. Barr [14] revisited the species and accepted it in “*Lepteutypa”* which is closely related to *L. cupressi* by having 3-septate ascospores covered with mucilaginous sheath and their sizes. However, *L. cupressi* has been accepted as *Seiridium cupressi* [16,69].

*Lepteutypa biseptata* Petr., Sydowia 8 (1–6): 197 (1954)

Typus: Australia, New South Wales, on thin branches of *Daviesia latifolia* (Fabaceae), 16 February 1951, E. Gauba (holotype).

Notes: Petrak [12] introduced *Lepteutypa biseptata* from Australia which has clypeate like greyish-brown discoloration, perithecia single or aggregated (two perithecia), and 1–3-septate ascospores with a mucilaginous sheath.

*Lepteutypa cisticola* Ade, Bot. Jb. 142: 109 (1928)

Typus: Spain, Canary Island, Tenerif, Barranco Anadigo, on *Cistus monspeliensis* (Cistaceae), 27 May 1926, A. Ade (W, Lectotype).

Notes: *Lepteutypa cisticola* has 3–5 gregarious ascomata, asci with J+ apical ring and euseptate, with 3-septate (or occasionally two transverse and one oblique) ascospores [70]. Petrak [71] introduced *Adea canariensis* while suggesting it was possibly the anamorph of *L. cisticola*. However, Nag Raj and Kendrick [70] re-described *Seiridium canariense* (= *Adea canariensis*).

*Lepteutypa fusispora* Petr., Sydowia 7(5–6): 387 (1953)

Typus: USA, Hawaii, on thin branches of *Wisteria* sp. (Fabaceae), 14 January 1928, Volcano, Nr. 1131a (holotype).

Notes: *Lepteutypa fusispora* has more or less often aggregated (often paired) perithecia and 1-septate, straight or curved ascospores [11]. The author has noted that the specimen was young, and asci and ascospores often shrink, and *Diaporthe seposita* is often found on the same collection. Nag Raj and Kendrick [70] re-examined the type specimen and remarked that “*L. fusispora*” is not congeneric with *A fuckelii* based on the J+ asci and ascospores.

*Lepteutypa hederae* (Fuckel) Rappaz, Mycol. helv. 7(1): 160 (1995)

≡ *Amphisphaeria hederae* Fuckel, Jb. nassau. Ver. Naturk. 25-26: 304 (1871)

= *Anthostoma hederae* (Fuckel) Sacc., Syll. fung. (Abellini) 1: 301 (1882)

Typus: Switzerland, near Neuchatel, on dead, corticated branches of *Hedera helix* (Araliaceae), by xylotomy probably *Viburnum* sp. (Adoxaceae), Mar. 1867, P. Morthier (G00111726!; Herbier Fuckel 1894 ex Herb, holotype).

Notes: Rappaz [72] accommodated the species in *Lepteutypa*. Jaklitsch et al. [16] re-examined the holotype, which was reported in the protologue to grow on corticated branches of *Hedera helix* (but as revealed by xylotomy, the host is probably *Viburnum* sp.) from Switzerland. *Lepteutypa hederae* possesses consistently 3-septate ascospores, lacking a median euseptum and asci with a J− apical ring.

*Lepteutypa hexagonalis* Goh & K.D. Hyde, Mycol. Res. 101(1): 85 (1997)

Typus: Ecuador, Oriente, Napo Province, Rio Cuyabeno, Cuyabeno rainforest, on dead trunk of *Pinanga* sp. (Arecaceae), August 1993, K. D. Hyde (BRIP 23007, holotype).

Notes: Goh and Hyde [15] introduced *Lepteutypa hexagonalis* which is characterized by having single or in groups of two, deeply immersed ascomata with a distinct ostiole, cylindrical asci with a J+ apical ring and brown, 3-septate, ascospores with 6–7 longitudinal ridges.

*Lepteutypa podocarpi* (Butin) Aa, Sydowia 39: 1 (1987) [1986]

= *Keissleriella podocarpi* Butin, Sydowia 27(1–6): 273 (1975) [1973–1974]

Typus: Chile, La Unión, leaves of *Podocarpus nubigenus* (Podocarpaceae), 29 August 1968, H. Butin (holotype).

Notes: *Lepteutypa podocarpi* was introduced by Butin [73] from *Podocarpus nubigenus* in Chile. Based on similar ascospore septation and a pestaloid asexual morph, van der Aa [74] accepted this taxon in *Lepteutypa*.

*Lepteutypa sabalicola* (Ellis & G. Martin) M.E. Barr, Mycotaxon 46: 57 (1993)

= *Sphaeria sabalicola* Ellis & G. Martin, Am. Nat. 16: 810 (1882)

= *Leptosphaeria sabalicola* (Ellis & G. Martin) Sacc., Syll. fung. (Abellini) 2: LVII (1883)

= *Heptameria sabalicola* (Ellis & G. Martin) Cooke, Grevillea 18(no. 86): 32 (1889)

Typus: USA, Florida, on petioles of *Serenoa serrulata* (= *Sabal serrulata*, Arecaceae), Feb. 1881, Martin (holotype).

Notes: Barr [14] re-examined the type of the specimen on *Serenoa serrulata* from Florida. Ascomata are usually gregarious beneath a conspicuous blackened clypeus. Ascospores are verruculose under the narrow hyaline coating. Another collection from Florida on *Aralia spinosa* is identical in asci and ascospores, but the ascomata form beaks that protrude 330–449 beyond the blackened clypeus.

*Lepteutypa tropicalis* Dulym., Sivan., P.F. Cannon & Peerally, in Dulymamode, Cannon & Sivanesan, Mycol. Res. 105(2): 249 (2001)

Typus: Mauritius, Petrin, on dead basal leaves of *Pandanus rigidifolius* (Pandanaceae), 30 April 1996, R. Dulymamode, P52 (mycol. herb. Univ. Mauritius, holotype; IMI 376738, isotype).

Notes: Dulymamode et al. [75] introduced *Lepteutypa tropicalis* on the adaxial surface of dead fallen leaf bases of *Pandanus rigidifolius* and *P. palustris* from Mauritius.

*Lepteutypa ulmicola* (Ellis & Everh.) M.E. Barr, Mycotaxon 46: 57 (1993)

= Clypeosphaeria ulmicola Ellis & Everh., Proc. Acad. nat. Sci. Philad. 45: 138 (1893)

Typus: Canada, Ontario, on dead branches of *Ulmus* sp. (Ulmaceae), April 1892, Dearness, 1776 (NY, holotype).

Notes: Ascospores of *Lepteutypa ulmicola* have one median septum and two distosepta with strongly pigmented and irregularly roughened walls.

#### 3.3.4. Hymenopleella

*Hymenopleella* Munk, Dansk Bot. Ark. 15(no. 2): 89 (1953)

MycoBank: MB2416; Facesoffungi number: FoF08765

= *Dyrithiopsis* L. Cai et al., Mycologia 95: 912 (2003)

= *Neotruncatella* Hyang B. Lee & T.T.T. Nguyen, Fungal Diversity 80: 198 (2016)

= *Trochilispora* V.P. Abreu, A.W.C. Rosado & O.L. Pereira, Fungal Diversity: 96, 169 (2019)

Type species: *Hymenopleella hippophaëicola* Jaklitsch & Voglmayr, Persoonia 37: 96 (2016)

MycoBank: MB814829; Facesoffungi number: FoF08766

Typus: Austria, Niederösterreich, Gerasdorf, Marchfeldkanalweg, on twigs of *Hippophaë rhamnoides* (Elaeagnaceae), 12 August 2012, W. Jaklitsch (WU 32027, epitype); ex-type living culture CBS 140410.

Notes: *Hymenopleella* is a sexual morph genus and sexual-asexual connection was confirmed through phylogeny by Liu et al. [39] while synonymizing *Dyrithiopsis* and *Neotruncatella* under *Hymenopleella*. Jaklitsch et al. [16] epitypified the type species, *Hymenopleella hippophaëicola*, while Liu et al. [39] emended the generic description and provided updated phylogeny.

*Hymenopleella schefflerae* (V.P. Abreu, A.W.C. Rosado & O.L. Pereira) Samarak., Maharachch. & K.D. Hyde, comb. nov.

MycoBank: MB836133; Facesoffungi number: FoF08767

≡ *Trochilispora schefflerae* V.P. Abreu, A.W.C. Rosado & O.L. Pereira, in Hyde et al., Fungal Divers 96: 171–173 (2019)

Typus: Brazil, Minas Gerais, Paraopeba, Floresta Nacional de Paraopeba (FLONA-Paraopeba), on leaves of *Schefflera morototoni* (Araliaceae), 30 January 2016, V.P. Abreu & O.L. Pereira (VIC 44384, holotype); ex-type living culture COAD 2371.

For description, see Hyde et al., Fungal Divers 96: 1–242 (2019)

Notes: A BLASTn search of ITS and LSU sequences of *Trochilispora schefflerae* (COAD 2371) are most similar to *Hymenopleella* (= *Dyrithiopsis*) (Sporocadaceae). The LSU-ITS-RPB2 phylogeny in this study also revealed that *T. schefflerae* clustered with *Hymenopleella austroafricana* (CPC 21940) with high statistical support. When the genus was introduced in Amphisphaeriaceae, the phylogeny lacked sufficient taxa selection, i.e., they used taxa in Bartaliniaceae, Discosiaceae, Pestalotiopsidaceae, and Robillardaceae which are now synonymized under Sporocadaceae [16]. In addition, the genera used in Amphisphaeriaceae are now accepted in Sporocadaceae (*Monochaetia*, *Morinia*) and Sordariomycetes genera *incertae sedis* (*Ellurema*) [39]. The phylogenetic study in Hyde et al. [20] showed that *T. schefflerae* is closely related to *Hymenopleella hippophaeicola* (CBS 140410). However, *Trochilispora* was introduced as a new genus with an uncertain phylogenetic position in Amphisphaeriaceae [20]. *Trochilispora schefflerae* was introduced based on acervular conidiomata, a 3–5-celled, thickened, brown peridium, small conidiophores, discrete, annellidic conidiogenous cells and fusiform, straight or slightly curved, 3–4-septate conidia with medium brown central cells and hyaline to subhyaline end cells with an apical cell with a tubular, filiform, single, eccentric, unbranched, aseptate appendage and basal cell without a basal appendage. However, the photo plate shows a coelomycetous conidiomata and similar morphology to *Hymenopleella* (see Hyde et al. [20], Liu et al. [39]). *Trochilispora schefflerae* and *H. austroafricana* possess discrete, annellidic conidiogenous cells (3.5–11.5 × 1.5–3 μm vs. 4–11.5 × 1.5–3 μm) and septate conidia (3–4-septate, 13–21 × 3.5–5 μm vs. 3–5 mostly 4-septate, 15.5–× 4–5 μm). Conidia of *Trochilispora schefflerae* have longer apical appendages (2.5–7.5 μm) compared to those of *H. austroafricana* (1.5–4.5 μm). Moreover, the spores of *T. schefflerae* lack basal appendages, which may be present in *H. austroafricana*. Based on morphology and phylogeny, here we treat *Trochilispora* as a synonym of *Hymenopleella* and accordingly recombine *T. schefflerae* in *Hymenopleella*.

## 4. Discussion

Jaklitsch et al. [16] concluded that the taxonomy of *Lepteutypa* and related genera is still unclear. Our morpho-molecular study corroborated this fact and revealed that *Lepteutypa* is an ill-defined genus and cannot be clearly segregated from *Amphisphaeria*. Barr [76] provided a key for genera in Amphisphaeriaceae. *Amphisphaeria* and *Lepteutypa* are placed together with brown ascospores (end cells hyaline or lightly pigmented at times) and single or few ascomata beneath the clypeus. According to her concept, taxa with 1-septate, smooth-walled or ornamented ascospore bearing species were placed in *Amphisphaeria*, while 3-septate ascospores with brown terminal cells, lacking elongate appendages, distoseptate at times, a wall usually ornamented and surrounded by narrow mucilaginous sheath were placed in *Lepteutypa*. This means that the ascospore septation played a key role in distinguishing the two genera. Jaklitsch et al. [16] combined *Clypeosphaeria unisepta* with *L. unisepta* addressing the ascospores septation as an insignificant characteristic for generic delimitation. Samuels et al. [65] had previously mentioned that the number of septa in the ascospores is not taxonomically significant at the generic level.

Several other studies also show that intraspecific morphological variations are not sufficient for generic delimitation (e.g., Hirayama and Tanaka [77]; Jaklitsch et al. [78]; Hyde et al. [79]; Wijayawardene et al. [80]). *Amphisphaeria* and *Lepteutypa* species differ in eutypoid stromata and septation of ascospores in the protologues. However, we consider that these morphologies are not sufficient to distinguish these genera. Here, we synonymize *Lepteutypa* under *Amphisphaeria*.

## Figures and Tables

**Table 1:** List of taxa used in the current phylogenetic study and GenBank acc. nos of DNA sequences. Strains and corresponding sequences that were newly obtained are printed in **bold**.

Species	Code	GenBank Accession Numbers				References
		LSU	ITS	RPB2	TUB2	
*Achaetomium macrosporum*	CBS 532.94	KX976699	KX976574	KX976797	KX976915	[38]
*Amphisphaeria acericola*	MFLU 16-2479	MK640424	MK640423	-	-	[8]
*Amphisphaeria acericola*	MFLUCC 14-0842 *	MF614131	MF614128	-	-	[8]
***Amphisphaeria camelliae***	HKAS 107021 *^,a^	**MT756615**	**MT756621**	**MT789850**	**MT774368**	**This study**
***Amphisphaeria camelliae***	**MFLUCC 20-0122 ^b^**	**MT756616**	**MT756622**	**MT789851**	**MT774369**	**This study**
***Amphisphaeria curvaticonidia***	**HKAS 102288 ^a^**	**MT756618**	**MT756624**	**MT789853**	**-**	**This study**
***Amphisphaeria curvaticonidia***	MFLUCC 18-0620 *^,b^	**MT756617**	**MT756623**	**MT789852**	**-**	**This study**
*Amphisphaeria flava*	MFLUCC 18-0361 *	MH971234	MH971224	-	MK033638	[7]
*Amphisphaeria fuckelii*	WU 33555	KT949903	KT949903	-	-	[16]
*Amphisphaeria fuckelii*	CBS 140409 *	KT949902	KT949902	MH554918	MH554677	[16,39]
*Amphisphaeria mangrovei*	NFCCI-4247 *	MG844275	MG844283	-	-	[6]
***Amphisphaeria micheliae***	HKAS 107012 *^,a^	**MT756619**	**MT756625**	**MT789854**	**MT774370**	**This study**
***Amphisphaeria micheliae***	**MFLUCC 20-0121^b^**	**MT756620**	**MT756626**	**MT789855**	**MT774371**	**This study**
*Amphisphaeria neoaquatica*	MFLUCC 14-0045 *	MK835805	MK828607	-	-	[17]
*Amphisphaeria qujingensis*	KUMCC 19-0186	MN707567	MN707568	-	-	[9]
*Amphisphaeria qujingensis*	KUMCC 19-0187 *	MN556316	MN477033	-	-	[9]
*Amphisphaeria sambuci*	CBS 131707 *	KT949904	KT949904	MH554911	MH704632	[16,39]
*Amphisphaeria sambuci*	WU 33557	KT949905	KT949905	-	-	[16]
*Amphisphaeria sambuci*	WU 33558	KT949906	KT949906	-	-	[16]
*Amphisphaeria sorbi*	MFLUCC 13-0721 *	KP744475	KR092797	-	-	[5]
*Amphisphaeria thailandica*	MFLU 18-0794 *	MH971235	MH971225	MK033640	MK033639	[7]
*Amphisphaeria umbrina*	AFTOL-ID 1229	FJ176863	FJ176809	-	-	[40]
*Amphisphaeria uniseptata*	CBS 114967 *	MH554197	-	MH554878	MH554638	[39]
*Amphisphaeria yunnanensis*	KUMCC 19-0188 *	MN556306	MN477177	-	-	[9]
*Amphisphaeria yunnanensis*	KUMCC 19-0189	MN550992	MN550997	-	-	[9]
*Anungitea eucalyptorum*	CBS 137967	KJ869176	KJ869118	-	-	[41]
*Anungitea grevilleae*	CBS 141282	KX228304	KX228252	-	-	[42]
*Bagadiella lunata*	CBS 124762 *	GQ303300	GQ303269	-	-	[43]
*Bartalinia pini*	CBS 143891 *	MH554330	MH554125	MH555033	MH554797	[39]
*Bartalinia pondoensis*	CBS 125525 *	MH875078	MH863602	MH554904	MH554663	[39,40]
*Beltrania pseudorhombica*	CBS 138003 *	KJ869215	KJ869158	MH555032	-	[39,41]
*Beltrania rhombica*	CBS 123.58	MH869260	MH857718	MH554899	MH704631	[39,44]
*Beltraniella endiandrae*	CBS 137976 *	KJ869185	KJ869128	-	-	[41]
*Beltraniopsis neolitseae*	CBS 137974 *	KJ869183	KJ869126	-	-	[41]
*Castanediella acaciae*	CBS 139896 *	KR476763	KR476728	-	-	[45]
*Castanediella brevis*	KUMCC 18-0132 *	MH806358	MH806361	-	-	[46]
*Castanediella eucalypti*	CBS 139897 *	KR476758	KR476723	-	-	[45]
*Castanediella eucalypticola*	CBS 141317 *	KX228317	KX228266	-	KX228382	[42]
*Chaetomium elatum*	CBS 374.66	MH870466	KC109758	KF001820	KC109776	[38,44]
*Clypeophysalospora latitans*	CBS 141463 *	KX820261	KX820250	-	-	[47]
*Cylindrium corymbiae*	CBS 146087 *	MT223887	MT223792	MT223679	MT223732	[48]
*Cylindrium grande*	CPC 35403 *	MK876425	MK876384	MK876481	MK876502	[49]
*Heterotruncatella acacigena*	CBS 143880 *	MH554295	MH554084	MH554996	MH554756	[39]
*Heterotruncatella vinaceobubalina*	CBS 143897	MH554341	MH554139	MH555045	MH554812	[39]
*Hymenopleella hippophaeicola*	CBS 140410 *	KT949901	KT949901	MH554919	MH554678	[16,39]
*Hymenopleella polyseptata*	CBS 143887 *	MH554321	MH554116	MH555024	MH554789	[39]
*Hymenopleella schefflerae*	COAD 2371 *	MH084761	MH128360	-	MH231215	[20]
*Hymenopleella austroafricana*	CBS 143886 *	MH554320	MH554115	MH555023	MH554788	[39]
*Neophysalospora eucalypti*	CBS 138864 *	KP004490	KP004462	-	-	[50]
*Phlogicylindrium uniforme*	CBS 131312 *	JQ044445	JQ044426	MH554910	MH704634	[39,51]
*Phlogicylindrium eucalyptorum*	CBS 111680	KF251707	KF251204	KF252209	KF252698	[52]
*Plectosphaera eucalypti*	CBS 120063 *	DQ923538	DQ923538	-	-	[53]
*Robillarda roystoneae*	CBS 115445 *	MH874545	KR873254	MH554880	KR873317	[39,44,54]
*Seimatosporium rosae*	MFLUCC 14-0621 *	MH823070	LT853105	LT853153	LT853253	[3,55]
*Seiridium marginatum*	CBS 140403 *	MH878679	KT949914	LT853149	LT853249	[16,44,55]
*Sordaria fimicola*	CBS 723.96	MH874231	-	DQ368647	-	[44,56]

Types and authentic strains are indicated with (*). Codes with ^a^ and ^b^ are denoted holotypes and ex-type cultures, respectively. Missing sequences are indicated by (-). Newly generated sequences are in bold. Abbreviations: AFTOL-ID—Assembling the Fungal Tree of Life; CBS—Centraalbureau voor Schimmelcultures, Utrecht, The Netherlands; COAD—Culture Collection of the Universidade Federal de Viçosa, Brazil; CPC—Culture collection of Pedro Crous, housed at CBS; HKAS—The Herbarium of Cryptogams Kunming Institute of Botany Academia Sinica, Kunming, China; KUMCC—Kunming Institute of Botany Culture Collection, Kunming, China; MFLU—Mae Fah Luang University Herbarium, Chiang Rai, Thailand; MFLUCC—Mae Fah Luang University Culture Collection, Chiang Rai, Thailand; NFCCI—National Fungal Culture Collection of India, Agharkar Research Institute, India. WU—Herbarium of the Institute of Botany, University of Vienna, Austria.

## Supplementary Materials

The following are available online at https://www.mdpi.com/2309-608X/6/3/174/s1. Figure S1: ML tree revealed by RAxML from an analysis of the LSU-ITS matrix of the Amphisphaeriaceae and selected families of Amphisphaeriales.
