# Supplementary material for: Molecular Phylogeny and Morphology of Amphisphaeria (= Lepteutypa) (Amphisphaeriaceae)
Source: J Fungi (Basel). 2020 Sep 17;6(3):174. doi: 10.3390/jof6030174 (PMC7558453; doi:10.3390/jof6030174)
Supplement: Supplementary file 1 [file jof-06-00174-s001.pdf]

Supplementary material for

Molecular Phylogeny and Morphology of *Amphisphaeria* (= *Lepteutypa*) (Amphisphaeriaceae)

Milan C. Samarakoon [1,2,3,4,7], Sajeewa S. N. Maharachchikumbura [4], Jian-Kui (Jack) Liu [4], Kevin D. Hyde [3,5], Itthayakorn Promputtha [1,2,*] and Marc Stadler [6*]

[1] Department of Biology, Faculty of Science, Chiang Mai University, Chiang Mai 50200, Thailand; samarakoon_m@cmu.ac.th (M.C.S.)

[2] Research Center in Bioresources for Agriculture, Industry and Medicine, Chiang Mai University, Chiang Mai 50200, Thailand; itthayakorn.p@cmu.ac.th (I.P.)

[3] Center of Excellence in Fungal Research, Mae Fah Luang University, Chiang Rai 57100, Thailand; coe-fungal@mfu.ac.th

[4] School of Life Science and Technology, University of Electronic Science and Technology of China, Chengdu 611731, P.R. China

[5] Innovative Institute of Plant Health, Zhongkai University of Agriculture and Engineering, Haizhu District, Guangzhou 510225, P.R. China

[6] Department of Microbial Drugs, Helmholtz-Zentrum für Infektionsforschung GmbH, Inhoffenstrasse 7, 38124 Brunswick, Germany

[7] Graduate School, Chiang Mai University, Chiang Mai, 50200, Thailand

* Correspondence: itthayakorn.p@cmu.ac.th (I.P.); marc.stadler@helmholtz-hzi.de (M.S.); Tel.: +66 833344392 (I.P.); +49-531-6181-4240 (M.S.)

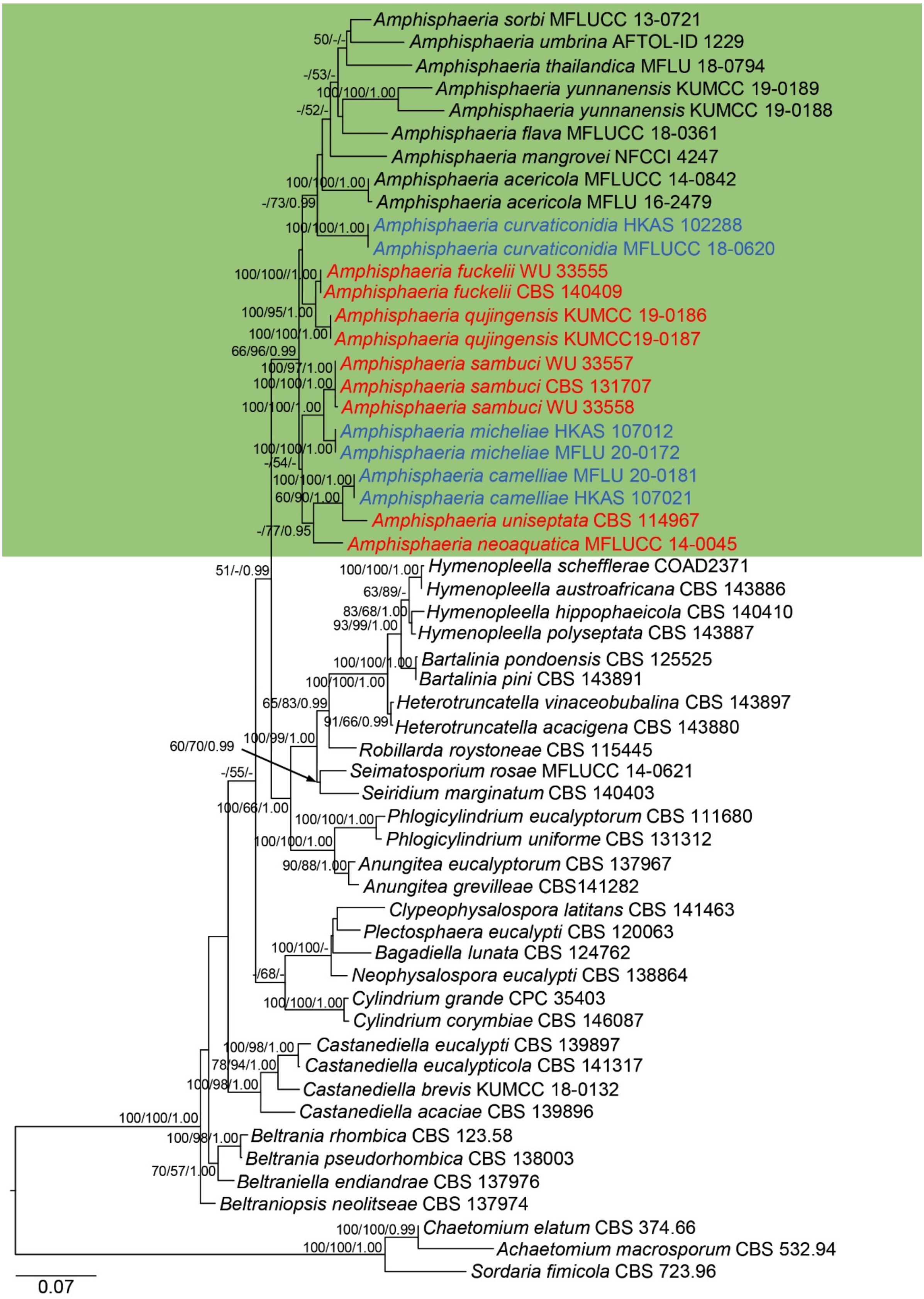

Figure S1. ML tree revealed by RAxML from an analysis of the LSU-ITS matrix of the Amphisphaeriaceae and selected families of Amphisphaeriales. Bootstrap supports (≥ 50%) of MP and ML and the posterior probability values (≥ 0.9) of BI analyses are

indicated above or below the respective branches. Newly generated sequences are in blue and type strains are in bold. The tree is rooted to *Achaetomium macrosporum, Chaetomium elatum* and *Sordaria fimicola* (Sordariales). The scale bar represents the expected number of nucleotide substitutions per site. Novel combinations are in red. Newly introduced taxa are in blue.
